# Radiotherapy-Associated Long-term Modification of Expression of the Inflammatory Biomarker Genes *ARG1, BCL2L1*, and *MYC*

**DOI:** 10.3389/fimmu.2017.00412

**Published:** 2017-04-10

**Authors:** Grainne Manning, Aleš Tichý, Igor Sirák, Christophe Badie

**Affiliations:** ^1^Cancer Mechanisms and Biomarkers Group, Centre for Radiation, Chemical and Environmental Hazards, Radiation Effects Department, Public Health England, Oxfordshire, UK; ^2^Department of Radiobiology, Faculty of Military Health Sciences in Hradec Králové, University of Defence, Brno, Czechia; ^3^Biomedical Research Centre, University Hospital Hradec Králové, Hradec Králové, Czechia; ^4^Clinic of Oncology and Radiotherapy, University Hospital Hradec Králové, Hradec Králové, Czechia

**Keywords:** radiation, inflammation, toxicity, biomarker, transcription

## Abstract

Ionizing radiation (IR) exposure of cells *in vitro* and *in vivo* triggers a complex cellular response among which modifications of gene expression have been consistently reported. Nevertheless, little is currently known about the transcriptionally responsive genes which play a role in the inflammation response. In order to improve our understanding of such transcriptional response to radiation *in vivo*, we simultaneously monitored the expression of 249 genes associated with the inflammation response over the course of the radiotherapy treatment in blood of patients treated for endometrial or head and neck cancer. We have identified genes whose transcriptional expression is either upregulated (*ARG1, BCL2L1*) or downregulated (*MYC*) several fold *in vivo*. These modifications were consistently detected across patients and further confirmed by quantitative real-time polymerase chain reaction (QRT-PCR); they were specifically significant toward the end of the radiotherapy treatment, 5 weeks following the first radiation fraction and more pronounced in endometrial patients (respectively, 2.9, 4.1, and 1.8 times). Importantly, in an attempt to correlate expression levels with normal tissue reaction to IR, we also identified three other genes *CD40, OAS2*, and *CXCR1* whose expression level fluctuations during radiotherapy were more pronounced in patients developing late normal tissue responses to curative radiotherapy after the end of the radiotherapy treatment. Overall, we identified inflammation-associated genes which are promising biomarkers of IR exposure and susceptibility to radiation-induced toxicity.

## Introduction

Humans are exposed to ionizing radiation (IR) from both environmental and medical sources. At the cellular level, IR has cytotoxic effects and is a physiologically important stress inducing a large range of DNA lesions (1) to which cells respond by the activation of multiple signaling pathways. DNA damage triggers the DNA-damage response, a complex network that regulates cell cycle, proliferation, and cell death. DNA repair is activated to ensure that the lesions are repaired efficiently and accurately with minimal impact on genome stability (2). Cellular exposure to IR also results in complex alterations in gene expression (3, 4), a fundamental mechanism of great importance for cells in order to execute their functions. Many investigations on global gene expression profiling of IR-exposed whole blood samples have identified genes associated with the DNA-damage response. Among others, we found many genes activated by the transcription factor p53 (encoded by the gene TP53) *via* the nuclear ataxia-telangiectasia mutated gene, the sensor of double-strand breaks (5–7), and some are promising biomarkers of radiation exposure for biological dosimetry purposes, e.g., *PCNA, DDB2, FDXR, CCNG1*, and *MDM2* (8–10).

Over recent years, a greater understanding has been obtained of the transcriptional response in cells and expression of specific genes can depend on radiation dose (11–13), dose rate (14, 15), radiation quality (16), and lapse between stress and analysis (17, 18). The level of dose also plays an important role. Low doses of IR induce genes in a linear dose-dependent manner (7) but specific immune responses were detected after low doses in whole blood, showing the involvement of both innate and adaptive immunity (19). Interestingly, the first mammalian radiation-induced protein-coding gene, i.e., tumor necrosis factor (TNF) was reported in the late 1980s (20). An increase in TNF-alpha (TNF-α) mRNA is accompanied by the increased production of TNF-α protein which is a mediator of the cellular immune response. For example, TNF-α acts directly on vascular endothelium to increase the adhesion of leukocytes during the inflammatory process (21). In mammalian cells, IR elicits a multi-layered signaling response by activating many pro-survival pathways and key transcription factors (22). Among them, IR transiently activates the nuclear factor kappa B (NF-κB), a ubiquitous transcription factor that regulates gene expression profile of multiple genes. Importantly, NF-κB has a central role in immune and inflammatory responses because it regulates the expression of pro-inflammatory cytokines and chemokines such as TNF-α (23). Although the aforementioned gene is directly involved in the inflammation process and was one of the first genes to be reported as being transcriptionally activated by radiation, only a few publications specifically studied inflammation-associated transcription modifications *in vitro* (19, 24, 25).

Inflammation also plays a key role in the response to radiation *in vivo* (26). As transcription factors regulate a wide spectrum of genes involved in inflammation, for example, NF-κB and p53 coregulate the induction of pro-inflammatory genes in primary human monocytes and macrophages (27), we decided to investigate IR exposure-associated transcriptional changes in an attempt to unravel the inflammation responses *in vivo* in human peripheral blood leukocyte (PBL) and platelets samples of patients undergoing radiotherapy treatment. Blood samples collected from endometrial and head and neck cancer patients treated by radiotherapy were analyzed at baseline and after the first, second, and last delivered dose (1.8 and 2 Gy, respectively). We investigated early and long-term chronic exposure effects on gene expression. Acute toxicity grading was evaluated as the worst grade of toxicity recorded during the treatment or up to 3 months after the end of treatment and late toxicity grading was evaluated as the worst grade of symptoms, persisting more than 3 months after the end of the treatment (see Materials and Methods for details). Moreover, we assessed interindividual variability in response among patients as some of them experienced toxic side effects of the radiotherapy treatment. Quantitative real-time polymerase chain reaction (QRT-PCR) was used to validate results obtained with the digital technology nCounter Analysis System, successfully used in the past to identify radiation-responsive genes in PBLs (28). Results for both techniques showed good correlation for all genes with *R*^2^ values ranging from 0.82 and 0.98.

## Materials and Methods

### Patient Radiotherapy Fractions and Radiation Toxicity Grading

Only cancer patients with no previous chemo- or radiotherapy were enrolled in the study. Patient ages ranged from 52 to 81 of which 7 head and neck patients were male, 1 head and neck patient was female, and with the 10 endometrial patients being female. The areas of radiation exposure for each cancer treatment and the prescribed dose for each patient listed in Table 1. Blood samples from 10 endometrial cancer patients and 8 head and neck cancer patients were collected into PAXGene tubes before radiotherapy treatment and at different times post-exposure as shown in Table 2. Both patient subgroups were treated for the same tumor localization in order to prevent the variability usually observed among patients treated with radiotherapy and to allow the corresponding roles of the size of irradiation field and of the dose rate to be studied. Blood from endometrial and head and neck cancer patients was taken pre-exposure, 24 h after the 1st fraction, 24 h after the 2nd fraction, and 24 h after the 25th fraction.

**Table 1:** **List of endometrial and head and neck cancer patients and their prescribed dose, dose per fraction, and calculated volume of blood irradiated**.

Category	Patient code	Prescribed dose (Gy)	Dose per fraction (Gy)	Mean-irradiated blood volume (dm^3^)
Endometrial cancer patients	E1–E10	45	1.8	1.1
Head and neck cancer patients	N2	50	2	0.5
	N1, N3	60	2	
	N8, N9	66	2	
	N4, N5, N7	70	2.1	

**Table 2:** **List of cancer patients and their recorded acute and late toxicity grades according to RTOG/EORTC late radiation morbidity criteria**.

Cancer patients	Patient code	Tumor grade	Sample taken	Acute toxicity	Late toxicity	Late toxicity location
Endometrial cancer patients	E1	2	Pre-exposure, 24 h, 48 h, 5 weeks	Grade 2	Grade 1	Intestinal (diarrhea)
	E2	2	Pre-exposure, 24 h, 48 h, 5 weeks	Grade 1	None	
	E3	1	Pre-exposure, 24 h, 48 h, 5 weeks	Grade 2	Grade 1	Intestinal (diarrhea)
	E4	1	Pre-exposure, 24 h, 48 h, 5 weeks	Grade 2	Grade 1	Intestinal (diarrhea)
	E5	3	Pre-exposure, 24 h, 48 h, 5 weeks	Grade 1	Grade 1	Intestinal (diarrhea)
	E6	2	Pre-exposure, 24 h, 48 h, 5 weeks	Grade 2	Grade 1	Intestinal (diarrhea)
	E7	2	Pre-exposure, 24 h, 48 h, 5 weeks	Grade 3	Grade 4	Intestinal (rectovaginal fistula)
	E8	2	Pre-exposure, 24 h, 48 h, 5 weeks	Grade 2	Grade 1	Intestinal (diarrhea)
	E9	1	Pre-exposure, 24 h, 48 h, 5 weeks	Grade 1	Grade 3	Bone (sacral plexopathy)
	E10	2	Pre-exposure, 24 h, 48 h, 5 weeks	Grade 2	None	
Head and neck cancer patients	N1	2	Pre-exposure, 24 h, 48 h, 5 weeks	Grade 1	Grade 1	Subcutaneous/mucosal
	N2	3	Pre-exposure, 24 h, 48 h, 5 weeks	Grade 1	Early death^a^	
	N3	3	Pre-exposure, 24 h, 48 h, 5 weeks	Grade 1	Early death^a^	
	N4	2	Pre-exposure, 24 h, 48 h, 5 weeks	Grade 2	Grade 2	Subcutaneous/mucosal
	N5	2	Pre-exposure, 24 h, 48 h, 5 weeks	Grade 2	Grade 3	Subcutaneous/mucosal
	N7	3	Pre-exposure, 24 h, 48 h	Grade 2	Grade 2	Subcutaneous/mucosal
	N8	2	Pre-exposure, 24 h, 48 h	Grade 1	Grade 1	Subcutaneous/mucosal
	N9	3	Pre-exposure, 24 h, 48 h	Grade 1	Grade 1	Subcutaneous/mucosal

*Patients with the highest toxicity grades (grades 3 and 4) are highlighted with a solid line*.

*^a^The patients N2 and N3 died due to rapid progression of the cancer disease and not due to radiation toxicity (i.e., grade 5 or so-called “death directly related to radiation late effects”)*.

Side effects of treatment such as toxicity were also recorded for each patient (Table 2). Acute toxicity grading was evaluated as the worst grade of toxicity recorded during the treatment or up to 3 months after the end of the treatment—CTCAE v. 4.0 grading system was used as described in Table 3. The full definition of the grading system can be found at the RTOG website.^1^ Late toxicity grading was evaluated as the worst grade of symptoms, persisting more than 3 months after the end of the treatment—RTOG/EORTC late radiation toxicity scheme (29) was used.

**Table 3:** **List of CTCAE v. 4.0 grading system used for acute toxicity grading and RTOG grading system used for late toxicity grading, including description of the grades in relevant locations**.

Toxicity grade	CTCAE v. 4.0	RTOG
Grade 1	Mild pain	Intestine: mild diarrhea, cramping, bowel movements five times daily, slight rectal discharge, or bleeding
		Subcutaneous/mucous membrane: slight induration, loss of subcutaneous fat, slight atrophy, and dryness
Grade 2	Moderate pain	Subcutaneous/mucous membrane: moderate fibrosis and moderate atrophy
Grade 3	Severe pain	Bone: severe pain, tenderness, complete arrest of bone growth, and dense bone sclerosis
		Subcutaneous/mucous membrane: severe induration, loss of subcutaneous tissue, marked atrophy, and complete dryness
Grade 4	Life threatening	Intestine: necrosis, perforation, and fistula
Grade 5	Death	Death

### Patient Blood Sampling

Blood samples were collected from the radiotherapy-treated cancer patients in PAXGene tubes according to the manufacturers’ protocol (Qiagen, PreAnalytiX GmbH, Hilden, Germany). The tubes were kept at RT for 2 h before being frozen at −20°C. RNA was extracted from the samples using the PAXGene Blood miRNA Kit (Qiagen, PreAnalytiX GmbH, Hilden, Germany) according to the manufacturers’ protocol. RNA quantity was assessed by Nanodrop ND2000 (Nanodrop, Wilmington, DE, USA), and RNA quality was assessed by Tapestation 2200 (Agilent Technologies, CA, USA).

### nCounter Analysis

Samples were analyzed by the nCounter Analysis System (NanoString Technologies^®^, Inc., Seattle, WA, USA) according to the manufacturers’ guidelines. The nCounter Analysis System utilizes a novel digital color-coded barcode technology that is based on direct multiplexed measurement of gene expression. The technology uses molecular “barcodes” and single-molecule imaging to detect and count hundreds of unique transcripts in a single reaction. The RNA sample was hybridized overnight in solution with the set of target-specific biotinylated capture probes and barcode containing reporter probes. The tubes were then covered and incubated at 65°C for 12–18 h in a thermocycler. The PrepStation collected hybridized probe/target complexes while washing away unhybridized probes. The washed complexes were then added to a cartridge containing a streptavidin-derivatized surface, which anchored the biotinylated capture probe end. The complexes were stretched and aligned by applying an electrical field to the immobilized complexes; the reporter (barcode)-containing end was anchored during this process with a second biotin-containing oligonucleotide. To count the molecules, the cartridges containing the immobilized, aligned barcodes were placed in the Digital Analyzer. The nCounter Digital Analyzer counted individual fluorescent barcodes which are composed of seven spots made up of four colors specific for the gene of interest. It imaged each cartridge and using proprietary image analysis software, counted the individual barcodes across the surface. Data were collected in the form of a text file, containing a list of gene names and number of times the barcode for that gene is detected, providing a direct count of the number of transcripts. The raw code count data from the nCounter Analysis System were first normalized and background corrected using a standard curve constructed from spike-in controls. The molecular counts were normalized to internal controls and reference genes according to Geiss et al. (30). The samples were run using 90 ng RNA per sample on the Human Inflammation V2 panel, which consists of 249 genes and scanned at 555 field of view (FOV). FOV is the area of the cartridge surface which is imaged by the Digital Analyzer with 555 FOV providing the most detailed scan. The raw code count data were first normalized and background corrected using a standard curve constructed from spike-in controls. The molecular counts were normalized to internal controls and references genes according to Geiss et al. (30). Candidate genes that were selected were those that showed a significant upregulation in comparison to the control (*t*-test, *p* < 0.05).

### Quantitative Real-time Polymerase Chain Reaction

Reverse transcriptase reactions were performed using High Capacity cDNA Reverse transcription kit (Applied Biosystems, FosterCity, CA, USA) according to the manufacturer’s protocol with 350 ng of total RNA. QRT-PCR was performed using Rotor-Gene Q (Qiagen, Hilden, Germany). All reactions were run in triplicate using PerfeCTa^®^ MultiPlex qPCR SuperMix (Quanta Biosciences, Inc., Gaithersburg, MD, USA) with primer and probe sets for target genes at 300 nM concentration each. 3′6-Carboxyfluorescein (FAM) and CY5 (Eurogentec Ltd., Fawley, Hampshire, UK) were used as fluorochrome reporters for the double dye probes analyzed in multiplexed reactions with between two genes per run including the control. When validating primer and probes sets, the efficiencies were analyzed when the primer and probe sets were run separately and when ran together in a multiplex reaction to check for interference as per QMRT-PCR guidelines (31). The primer sequences for QRT-PCR analysis were *HPRT* F: 5′ TCAGGCAGTATAATCCAAAGATGGT 3′, R: 5′ AGTCTGGCTTATATCCAACACTTCG 3′, probe: 5′ CGCAAGCTTGCTGGTGAAAAGGACCC 3′; *MYC* F: 5′ CTTGTACCTGCAGGATCTGA 3′, R: 5′ GTCGAGGAGAGCAGAGAATC 3′, probe 5′ CGCCCAAGTCCTGCGCCTCG 3′. Cycling parameters were 2 min at 95°C, then 45 cycles of 10 s at 95°C and 60 s at 60°C. Data were collected and analyzed by Rotor-Gene Q Series Software. Gene target Ct (cycle threshold) values were normalized to a hypoxanthine-guanine phosphoribosyltransferase 1 (*HPRT1*) internal control. Ct values were converted to transcript quantity using standard curves obtained by serial dilution of PCR-amplified DNA fragments of each gene. The linear dynamic range of the standard curves covering six orders of magnitude (serial dilution from 3.2 × 10^−4^ to 8.2 × 10^−10^) gave PCR efficiencies between 93 and 103% for each gene with *R*^2^ > 0.998. Relative gene expression levels after irradiation were determined.

SYBRGreen PCR was performed using Rotor-Gene Q (Qiagen, Hilden, Germany). All reactions were run in triplicate using PerfeCTa SYBR^®^ Green SuperMix (Quanta Biosciences, Inc., Gaithersburg, MD, USA) with primer sets for target genes at 500 nM concentration each. Cycling parameters were 2 min at 95°C, then 40 cycles of 10 s at 95°C and 60 s at 60°C. Data were collected and analyzed by Rotor-Gene Q Series Software. Fold of change values were calculated using the delta–delta Ct method. The primer sequences for SYBRGreen analysis were *HPRT* F: 5′ TCAGGCAGTATAATCCAAAGATGGT 3′, R: 5′ AGTCTGGCTTATATCCAACACTTCG 3′; *ARG1* F: 5′ CCACCTAAGTAAATGTGGAAAC 3′, R: 5′ ACCAAGAGGGAATTTGTAGAG 3′; *BCL2L1* F: 5′ GGCTCTCTGCTGTACATATT 3′, R: 5′ GCAGCTCCTCACACATAA 3′; *CD40* F: 5′ GCAGGAGACTGGCTAAATAA 3′, R: 5′ CTGTGTACCCTTCCAGAAC 3′; *OAS2* F: 5′ CTGGGTTCACAGATCTTTCT 3′, R: 5′ GTTCTTGACCTTTGGGTATCT 3′; *CXCR1* F: 5′ GTCTGCTGGAGACATTGAG 3′, R: 5′ GGGTTCTTGTGGCATAGAT 3′.

A primer-probe design and a SYBR green design were used in order to produce results quickly. A primer-probe design was used for the gene *MYC* as it was already available in our lab. SYBR green was used for the other new genes identified by nCounter to provide confirmation of the results.

### Statistical Analysis

Statistical analysis of the biological data was performed using Minitab and Stata. Data points represent the mean ± SEM. *p* Values ≤ 0.05 were considered statistically significant. The data were tested for normal distribution. Parametric (*t*-test) and non-parametric (Mann–Whitney, Kruskal–Wallis) tests were used to test nCounter results for significance of candidate genes. Kruskal–Wallis tests were performed to test for significance of SYBR green QPCR results. *p*-Trend tests were performed using the software Stata to test for significance of dose-to-gene associations of *BCL2L1* and *OAS2*.

## Results

### nCounter Analysis

**Figure 1:**
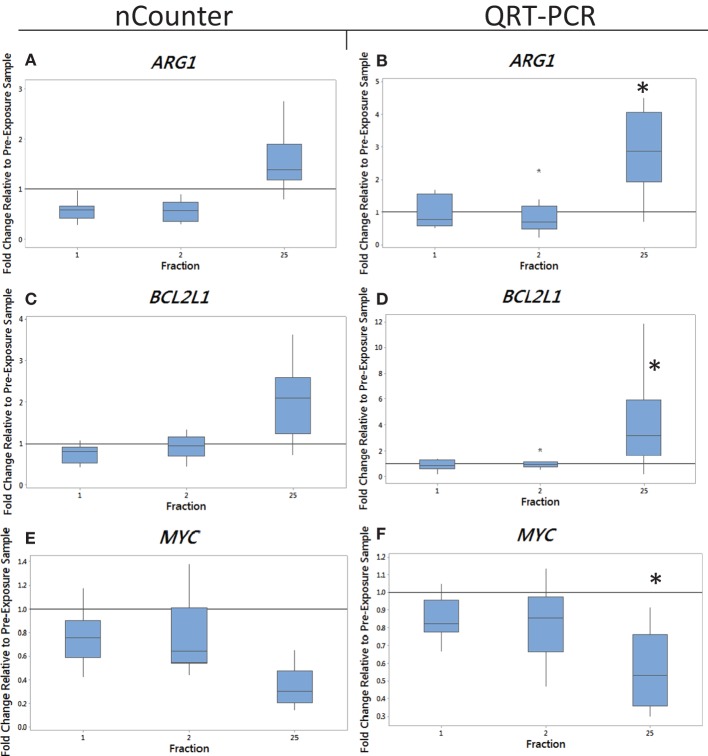
**The box plot shows the fold change in expression of the genes *ARG1* (A,B), *BCL2L1* (C,D), and *MYC* (E,F) in endometrial cancer patients 24 h after the 1st fraction, 24 h after the 2nd fraction, and 24 h after the 25th fraction**. The box plot is composed of a rectangular box representing the middle 50% of the data, the median value indicated by the horizontal line inside the box, lines representing the upper and lower 25% of the distribution, and outliers indicated by asterisks. Expression was measured using the nCounter (left) and QRT-PCR (right) analysis. Fold changes in expression compared to non-irradiated blood (and relative to *HPRT* gene). Significance was calculated using the Kruskal–Wallis test where *p < 0.05.

**Figure 2:**
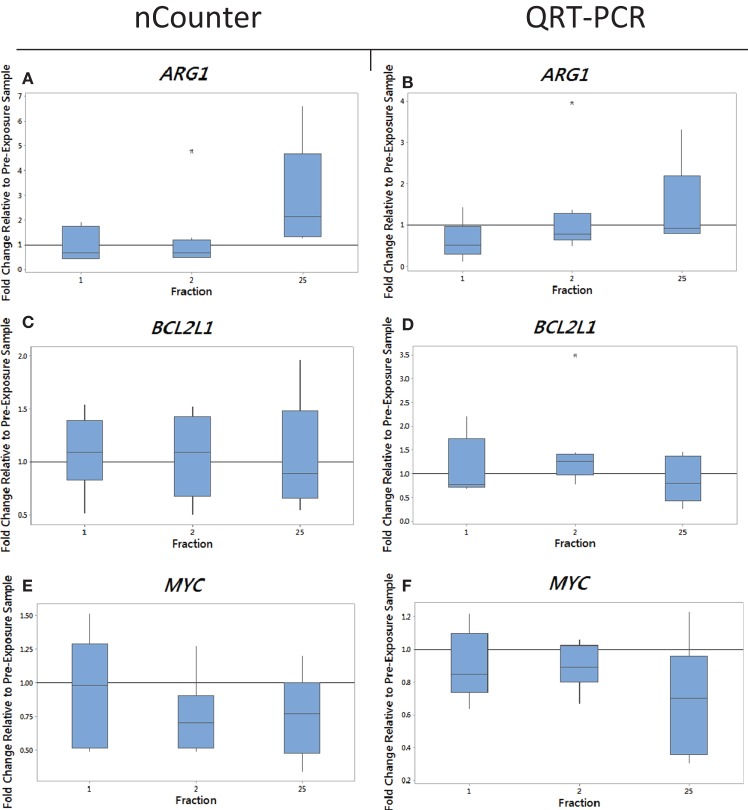
**The box plot shows the fold change in expression of the genes *ARG1* (A,B), *BCL2L1* (C,D), and *MYC* (E,F) in head and neck cancer patients 24 h after the 1st fraction, 24 h after the 2nd fraction, and 24 h after the 25th fraction**. The box plot is composed of a rectangular box representing the middle 50% of the data, the median value indicated by the horizontal line inside the box, lines representing the upper and lower 25% of the distribution, and outliers indicated by asterisks. Expression was measured using the nCounter (left) and RT-PCR (right) analysis. Fold changes in expression compared to non-irradiated blood (and relative to *HPRT* gene).

Blood from endometrial and head and neck cancer patients was taken pre-exposure, 24 h after the 1st fraction, 24 h after the 2nd fraction, and 24 h after the 25th fraction. Using the nCounter, we analyzed the transcriptional expression level of 249 genes associated with the inflammation process. Candidate genes were selected that showed a significant upregulation in comparison to the control (*p* < 0.05). From the inflammation panel, comparing blood samples obtained before and after the first or second fraction, we did not identify genes whose expression was consistently and significantly modified by radiation exposure (data not shown). To the contrary, a significant modification of expression after radiation exposure was detected at the last time point. Importantly, three genes were identified from the nCounter analysis that showed a modification in expression at day 35 (5 weeks following the first fraction, 24 h following the last fraction) as shown in Figures 1A,C,E (endometrial cancer patients) and Figures 2A,C,E (head and neck cancer patients). Two genes, *ARG1* and *BCL2L1* were upregulated while *MYC* was downregulated. These results were then confirmed by QRT-PCR analysis.

### Quantitative PCR Analysis

We monitored previously validated radiation-responsive genes to confirm that IR exposure could be detected in PBL. The majority of genes investigated responded rapidly to radiation exposure, reaching a peak of expression between 24 h after the first fraction (day 1) and 24 h after the second fraction (day 2) (data not shown). Mean gene expression values of nCounter plotted against QRT-PCR data showed good agreement between both methods with *R*^2^ values ranging from 0.90 to 0.99 for endometrial samples and ranging from 0.5 to 0.97 for head and neck samples for genes showing a change in expression (data not shown). The gene *ARG1* was upregulated at 5 weeks after fractionated therapy in 9 out of the 10 endometrial cancer patients (fold of change ranging from 0.7 for patient E4 up to 4.5-fold increase in expression for patient E6) (Figure 1B). This increase was also evident in head and neck cancer patients, but to a lower extent with fold changes of 3.3 and 1.8 in patients N1 and N2 with the rest showing no increase in expression at 5 weeks (Figure 2B). The gene *BCL2L1* showed a large variation in expression among endometrial cancer patients at 5 weeks with an 11.8-fold increase for patient E2 while other patients showed no modification of expression (Figure 1D). The expression of *BCL2L1* in head and neck cancer patients was low reaching 1.5-fold increase at week 5 for patient N4 but the remaining patients showing no increase in expression (Figure 2D). To the contrary, the gene *MYC* was consistently and gradually downregulated in both endometrial (Figure 1F) and head and neck cancer patients (Figure 2F) from the first time point (1 day post-first fraction) to the last one (5 weeks) where the downregulation became significant for the endometrial patients. At this late time point, *MYC* was downregulated 1.8-fold on average in endometrial cancer patient samples and showed a 1.5-fold downregulation in head and neck cancer patients. As with the other genes, this response was stronger in the endometrial cancer patients.

### Toxicity Analysis

**Figure 3:**
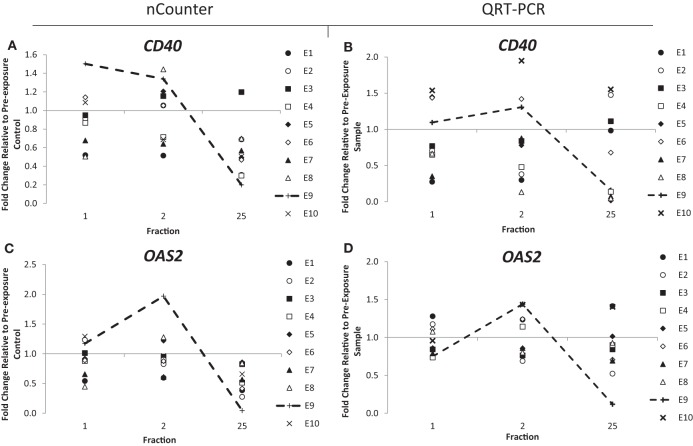
**Fold change in expression of the genes *CD40* (A,B) and *OAS2* (C,D) in endometrial cancer patients 24 h after the 1st fraction, 24 h after the 2nd fraction, and 24 h after the 25th fraction**. Expression was measured using the nCounter (left) and RT-PCR (right) analysis. Fold changes in expression compared to non-irradiated blood (and relative to *HPRT* gene).

**Figure 4:**
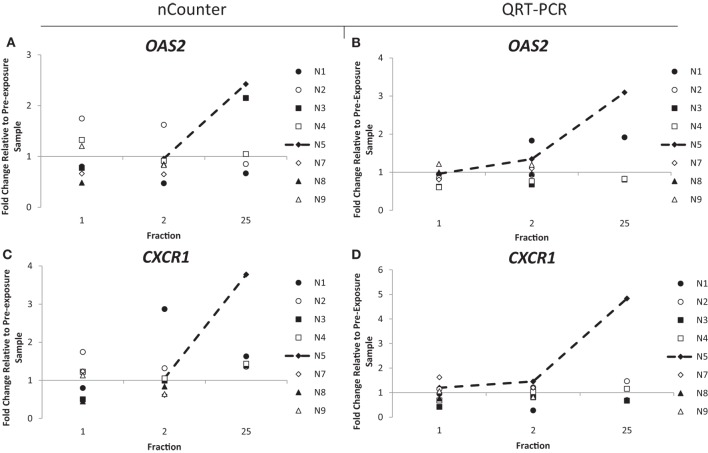
**Fold change in expression of the genes *OAS2* (A,B) and *CXCR1* (C,D) in head and neck patients 24 h after the 1st fraction, 24 h after the 2nd fraction, and 24 h after the 25th fraction**. Expression was measured using the nCounter (left) and RT-PCR (right) analysis. Fold changes in expression compared to non-irradiated blood (and relative to *HPRT* gene).

Out of the 10 endometrial cancer patients, 1 of them, patient E7, recorded the highest level of acute (grade 3) and late toxicity of grade 4 (Table 2) diagnosed as a rectovaginal fistula. Late stage toxicity was also identified in patient E9 who had painful sacral plexopathy. In the head and neck cancer patients, the highest toxicity level of grade 3 was recorded in patient N5 who experienced severe induration. We retrospectively searched for inflammation-associated genes whose expression would have been modified specifically in these three patients. Although we could not single out any gene with a specific up- or downregulation for patient E7, who had the highest late toxicity grade, the nCounter analysis identified two genes, *CD40* and *OAS2*, following the same pattern of expression with a slight increased expression of 1.3- and 1.4-fold in the endometrial cancer patient E9 at 48 h (Figures 3A,C). By the end of the radiotherapy treatment, the expression levels were inverted and a clear downregulation of sixfold and eightfold could be seen. This was confirmed by QRT-PCR analysis (Figures 3B,D). We then analyzed the data for patient N5. Of importance, the pattern of expression was different from patient E9, the nCounter analysis also identified *OAS2* as well as another gene *CXCR1*, showing an increased expression in the head and neck cancer patient N5 at 5 weeks (Figures 4A,C). This was confirmed by QRT-PCR analysis which showed an increase of 3- and 4.8-fold in expression at 5 weeks for *OAS2* and *CXCR1*, respectively (Figures 4B,D).

Head and neck cancer patients received treatments with 50–70 Gy. Dose versus fold change in gene expression per day was examined for patients receiving the different treatments. A slight dose–response with large variability was seen in only two of the genes (BCL2L1, OAS2) at 5 weeks (data not shown). A *p*-trend test was performed for these genes and although a trend was viable from the graphs, it was not statistically significant with a *p* value of 0.398 for *OAS2* and a *p* value of 0.257 for *BCL2L1*. The toxicity grading was analyzed by categorizing the gene expression response at the 25th fraction into two categories (grades 1 + 2 and grades 3 + 4). The grading of all patients could only be combined for the gene OAS2 due to the fact that different genes were identified for the two sets of cancer patients. For the gene OAS2 at the 25th fraction, a Mann–Whitney test was performed giving a *p* value of 0.475. Such analysis combining all types of late toxicities does not incorporate information on the localization of the toxicity which for patient E9 was in the bone while it was intestinal in E7 and mucosal in N5. Therefore, it may be more relevant to look at patients on a case by case basis where further details, such as location of toxicity, provide important information for transcriptional analysis. When the data were combined into two categories at the 25th fraction for the gene CD40 for endometrial cancer patients, this only resulted in six samples for the grade 1 category and two samples for the grade 3 and grade 4 categories. A Mann–Whitney test was performed giving a *p* value of 0.867. When the data were combined into two categories at the 25th fraction for the gene CXCR1 for head and neck patients, this again only resulted in two samples for the grade 1 and grade 2 categories and one sample for the grade 3 category. Unfortunately such analyses have limited significance with the small samples size (i.e., only one sample in the grade 3 category), and a *p* value could not be obtained. Due to the small samples sizes and lack of statistical analysis, these graphs were not included in the manuscript.

## Discussion

### Radiation-Induced Inflammatory Biomarkers

Biological research has been providing characterization and understanding of the complex actions of IR on biological processes. IR causes multiple types of damage to DNA but also the formation of reactive oxygen species which induce stress responses, inflammation, and release of cytokines, growth factors, and chemokines (32, 33). Immunological biomarkers of radiation-induced fibrosis and pneumonitis in cancer radiotherapy patients were reviewed by Sprung et al. (34). Nonetheless, radiation-induced inflammation-associated transcripts expressed in circulating PBL *in vivo* have not yet been explored. In particular, long-term effects have rarely been investigated and only the effects of acute long-term exposure on global gene expression patterns in irradiated human lymphocytes were reported (35). In this study, we looked specifically at transcripts of genes associated with inflammation and induced by IR and their correlation with long-term effects (i.e., after 3 months after the end of RT) such as radiation toxicity.

The use of the recently developed nCounter technology enabled us to screen 249 genes associated with inflammation simultaneously (Human Inflammatory V2 panel). We previously scan the expression of hundreds of genes following IR exposure using this technique successfully (28). Three genes were identified as radiation-induced inflammatory biomarkers in PBL *in vivo*. *ARG1* and *BCL2L1* show increased expression mainly toward the end (35 days) of the radiotherapy treatment while *MYC* shows a gradual increased downregulation with cumulative doses of radiotherapy treatment. For all three genes, this response was more pronounced in endometrial cancer patients where it becomes significant. Although we cannot provide an explanation, it is possible that this is a dose effect as the irradiated volume of body mass as well as circulating blood is higher in endometrial cancer patients in comparison to head and neck patients (see Table 1). The first gene, *ARG1*, catalyzes the hydrolysis of arginine to ornithine and urea and is expressed in macrophages. Interestingly its expression has been found upregulated *in vitro*, in primary monocytes-derived macrophages obtained from blood samples collected from patients before and after the first delivered 2 Gy radiotherapy dose in breast cancer patients (36). The authors found that the level of *ARG1* mRNA significantly correlated with higher grades of radiation-induced acute skin toxicities in early breast cancer patients. As discussed later, we also looked at acute and late radiation toxicity but could not find any correlation as the increase in *ARG1* expression was found in three patients on day 1 and in nine patients at 5 weeks where the level of expression becomes significantly different from basal expression. In our *in vivo* study, *ARG1* can be rather considered as a late biomarker of radiation exposure than a biomarker of radiation toxicity. The second gene we found to be significantly upregulated at 35 days, *BCL2L1*, is a member of the BCL-2 protein family, which are involved in a number of cellular functions such as apoptosis and regulation of the outer mitochondrial membrane channel (VDAC) opening. *BCL2L1* expression after radiotherapy has previously been investigated in prostate cancer patients undergoing external beam radiotherapy and found to be upregulated with increasing fatigue (37). Here, the expression of *BCL2L1* increases with time and at week 5 the gene is upregulated in nine patients; however, fatigue was not measured and so no comparisons to this factor can be made. *BCL2L1* has also been investigated as a predictive marker of radioresistance, however, there are conflicting reports. *BCL2L* expression in head and neck patients has shown to be associated with a favorable outcome in a study involving 400 patients (38) while another study associates *BCL2L1* expression with tumor recurrence (39). Finally, *MYC* is a well-known transcription factor that plays a central role in cancer development processes including cell proliferation, growth, and apoptosis. *MYC* has been previously upregulated in cases of radiation-induced angiosarcoma (40, 41) and glioblastoma, with its expression associated with longer overall survival (42) but here we see a strong and consistent downregulation in all endometrial cancer patients after radiotherapy.

We chose to analyze whole blood transcriptional responses as it was a simple and reliable protocol to collect and preserve RNA using specifically designed PAXgene tubes. PBL represents a complex combination of different cell types (neutrophils, lymphocytes, eosinophils, and basophils), allowing the study of a collective tissue response with neutrophils the most abundant (~60%) and short lived; therefore, a late change in transcription (i.e., 5 weeks) is unlikely to be from this specific subpopulation. On the other hand, it is not possible in this study to confirm that these radiation-induced modulations of expression are global or potentially cell-type specific. It is probable that results could be refined by sorting PBL subpopulations which may have a stronger transcriptional response to radiation. For instance, it has been shown that several biological responses in cluster of differentiation CD4+ cells could be more sensitive to low doses of radiation than CD56+ and CD8+ (43). When blood volumes are sufficient, further studies should be designed to isolate blood subpopulations before performing cell-type specific transcription analyses.

We and others have shown that gene expression analysis could be a powerful tool to predict radiation exposure for biological dosimetry purposes and such inflammatory gene expression signature (i.e., *ARG1, BCL2L1*, and *MYC*) may be useful not only for biodosimetric triage, as well as to monitor the progress of treatment and recovery.

### Radiation-Induced Toxicity Biomarkers

Normal tissue reactions to radiotherapy vary in severity among patients and cannot be accurately predicted, limiting treatment doses (44). The existence of heritable radiosensitivity syndromes [e.g., Ref. (45, 46)] suggests that normal tissue reaction severity is determined, at least in part, by genetic factors and these may be revealed by differences in gene expression. Transcriptional responses in lymphoblastoid cells can be used to understand the genetic basis for variation in human radiosensitivity (47), to assess interindividual susceptibility to DNA damaging agents for the prediction of therapeutic response to drugs (48), and to predict clinical outcome in human cancers (49). For example, we have previously shown that cyclin-dependent kinase inhibitor 1A (*CDKN1A*) transcriptional response associates with abnormal acute sensitivity to radiation treatment (50). Transcriptional responses to radiation (51, 52) also reported that changes of expression in a specific set of genes after *in vitro* irradiation of stimulated peripheral lymphocytes can, to some extent, successfully predict severe late reaction status.

Inflammation has a protective role and is a response mechanism involving multiple immune cells. Nevertheless, chronic inflammation is also associated with the development of chronic diseases such as radiation toxicity. In this study, we also searched for differences in gene expression discriminating individuals with marked responses with the aim to identify potential biomarkers of radiation toxicity that would facilitate normal tissue response prediction.

Three genes, *CD40, OAS2*, and *CXCR1*, were identified as potential biomarkers of normal tissue toxicity in cancer patients after radiotherapy. In endometrial cancer patients, we observed by simple visual screening that the expression of *CD40* and *OAS2* was particularly variable in patient E9 at the different time points studied although the number of patients studied here didn’t allow us to conclude in terms of statistical significance. *CD40* is a member of the TNF-receptor superfamily, which is involved in mediating a number of inflammatory processes with interference of the CD40–CD40 ligand. Interestingly, earlier work also reported a reduction of expression in radiation-induced lung toxicity in mice (53). The second gene, *OAS2*, is a member of the 2–5A synthetase family which is involved in the immune response to viral infections. Expression of *OAS2* has been suggested as a biomarker for disease and it has been reportedly upregulated in psoriasis and squamous cell carcinoma patients (54) and in mice in response to cigarette smoke and influenza virus (55).

Expression of *OAS2* was particularly inconstant in endometrial patient E9 and head and neck patient N5, both with reported toxicity side effects. In the endometrial cancer patients, expression of *OAS2* was upregulated at 48 h in patient E9, who was recorded as having the second highest late toxicity score of grade 3. This increase was followed by a drop of expression of a factor of 12 at 5 weeks, possibly indicating the beginning of an inflammation response and the painful sacral plexopathy the patient experienced. This upregulation was not seen in patient E7 who recorded the highest toxicity level of grade 4. Although we do not have an explanation for this, it might be due to the specificity of the response to the type of toxicity (patient E7 was diagnosed with rectovaginal fistula).

The expression of *OAS2* was also upregulated in the head and neck cancer patient N5, which reported the highest level of toxicity, grade 3. This upregulation was weak at day 2 but amplified after 5 weeks. We speculate that the later upregulation compared to patient E9 is possibly due to the smaller area treated for head and neck cancer patients and thus a threshold level of radiation exposure possibly needs to be achieved in order to upregulate this gene. More likely, the difference at 35 days, i.e., upregulation (N5) and downregulation (E9) might be linked to the nature of the tissue irradiated. Nevertheless, a shift in expression might be an indication of radiation toxicity occurring later.

Possibly, the clearest difference in expression between patients was for the gene *CXCR1*. It is a member of the G-protein-coupled receptor family, binding with high affinity to IL8 and mediating chemotaxis. With such a central role in the inflammatory response, *CXCR1* has been targeted for the development of pain-relieving drugs (56, 57). Similar to the gene *OAS2, CXCR1* was clearly upregulated in patient N5 at the 5-week time point with an increase in expression of nearly fivefold, again indicating that the inflammatory response in this patient can be detected by these genes.

As a general comment, we acknowledge that the tumors for which the patients were treated by radiotherapy may affect the basal level of expression of many inflammation genes analyzed in PBL in this study (58). Despite the fact that it might have affected the sensitivity of detection, it should not have affected the specificity, as the patient blood samples obtained 24 h before the beginning of the treatment were used to set-up the background level of expression of these genes. Potential confounding factors such as age at treatment and gender (for head and neck cancer patients) could not be investigated in this study due to the small sample size but would be of importance in future studies.

### Summary and Conclusion

To summarize, this study allowed the identification of three inflammatory-associated genes (*ARG1, BCL2L1*, and *MYC*) whose expression is consistently modified in cancer patients by the radiotherapy treatment more than a month after the beginning of the treatment and, although these results require confirmation and extension, it suggests the possibility of predicting the severity of radiation toxicity by monitoring the leukocyte mRNA levels of specific genes (for example, *CD40, OAS2*, and *CXCR1*). Identification of such biomarkers could improve treatment, comfort for the patient, and reduce side effects. These genes may possibly be used to identify patients who are at risk of developing severe toxicity and appropriate measures could be taken to reduce radiation toxicity in these patients. We have identified potential biomarkers of late toxicity in which expression was upregulated only after completion of radiotherapy, but before clinical signs could be detected. The changes in gene expression 24 h after the last radiotherapy fraction (25th) precede the late tissue reaction developed in patients E7 and N5. Upregulation of these biomarkers would not influence the indication or dose of radiation since it can be detected after the end of the treatment. However, more intensive surveillance and supportive care may be needed in patients with detected activity of these biomarkers after the treatment. Our findings are important for future radiation late morbidity understanding and may be a potential aim for targeting in late morbidity prevention.

In conclusion, this study demonstrates the importance of further exploration of the modifications of transcription in response to IR exposure in genes associated with an inflammation response and the immune system. In general, it has the potential to be a source of biomarkers allowing to complete the portfolio of identified mRNA transcripts for monitoring radiation exposure during radiotherapy on one hand and, perhaps more importantly, of radiation toxicity on the other one.

## Ethics Statement

This study was carried out in accordance with the recommendations The Code of Ethics of the World Medical Association—Declaration of Helsinki (approval no: 201401-S15P) with written informed consent from all subjects. All subjects gave written informed consent in accordance with the Declaration of Helsinki. The protocol was approved by the Ethical Committee of University Hospital in Hradec Kralove (Czechia).

## Author Contributions

All persons who meet authorship criteria are listed as authors, and all authors certify that they have participated sufficiently in the work to take public responsibility for the content, including participation in the concept, design, analysis, writing, or revision of the manuscript.

## Disclaimer

The authors alone are responsible for the content and writing of the paper. This report is work commissioned by the National Institute for Health Research. The views expressed in this publication are those of the authors and not necessary those of the NHS, the National Institute for Health Research, or the Department of Health.

## Conflict of Interest Statement

The authors declare that the research was conducted in the absence of any commercial or financial relationships that could be construed as a potential conflict of interest.

## Footnotes

^1^https://www.rtog.org/ResearchAssociates/AdverseEventReporting.aspx.
